# The Tomosynthesis Broken Halo Sign: Diagnostic Utility for the Classification of Newly Diagnosed Breast Tumors

**DOI:** 10.3390/tomography9060155

**Published:** 2023-10-24

**Authors:** Johannes Deeg, Michael Swoboda, Daniel Egle, Verena Wieser, Afschin Soleiman, Valentin Ladenhauf, Malik Galijasevic, Birgit Amort, Silke Haushammer, Martin Daniaux, Leonhard Gruber

**Affiliations:** 1 Department of Radiology, Medical University Innsbruck, Anichstraße 35, 6020 Innsbruck, Austria; johannes.deeg@i-med.ac.at (J.D.); valentin.ladenhauf@i-med.ac.at (V.L.); malik.galijasevic@i-med.ac.at (M.G.); b.amort@tirol-kliniken.at (B.A.); silke.haushammer@tirol-kliniken.at (S.H.); martin.daniaux@tirol-kliniken.at (M.D.); leonhard.gruber@i-med.ac.at (L.G.); 2 Department of Gynecology, Medical University Innsbruck, Anichstraße 35, 6020 Innsbruck, Austria; daniel.egle@i-med.ac.at (D.E.); verena.wieser@i-med.ac.at (V.W.); 3 Institute for Pathology, INNPath, University Hospital Tirol Kliniken, Anichstraße 35, 6020 Innsbruck, Austria; afschin.soleiman@innpath.at

**Keywords:** tomosynthesis, breast cancer, broken halo sign

## Abstract

Background: Compared to conventional 2D mammography, digital breast tomosynthesis (DBT) offers greater breast lesion detection rates. Ring-like hypodense artifacts surrounding dense lesions are a common byproduct of DBT. This study’s purpose was to assess whether minuscule changes spanning this halo—termed the “broken halo sign”—could improve lesion classification. Methods: This retrospective study was approved by the local ethics review board. After screening 288 consecutive patients, DBT studies of 191 female participants referred for routine mammography with a subsequent histologically verified finding of the breast were assessed. Examined variables included patient age, histological diagnosis, architectural distortion, maximum size, maximum halo depth, conspicuous margins, irregular shape and broken halo sign. Results: While a higher halo strength was indicative of malignancy in general (*p* = 0.031), the broken halo sign was strongly associated with malignancy (*p* < 0.0001, odds ratio (OR) 6.33), alongside architectural distortion (*p* = 0.012, OR 3.49) and a diffuse margin (*p* = 0.006, OR 5.49). This was especially true for denser breasts (ACR C/D), where the broken halo sign was the only factor predicting malignancy (*p* = 0.03, 5.22 OR). Conclusion: DBT-associated halo artifacts warrant thorough investigation in newly found breast lesions as they are associated with malignant tumors. The “broken halo sign”—the presence of small lines of variable diameter spanning the peritumoral areas of hypodensity—is a strong indicator of malignancy, especially in dense breasts, where architectural distortion may be obfuscated due to the surrounding tissue.

## 1. Introduction

Breast cancer is the most common malignancy in women, with an incidence of up to 92 per 100.000 women in highly developed countries, which is continually rising due to an increase in life expectancy [1]. Fortunately, up to 80% of early stage, non-metastatic cases can be cured [2]. Advanced breast cancer, defined by the presence of distant metastasis, is still considered incurable [2], underlining the importance of early detection and treatment.

While diagnostic imaging is the mainstay in curative and screening protocols, digital breast tomosynthesis (DBT) offers better detection rates by reconstructing a pseudo three-dimensional digital mammography thin section series from low-dose X-ray projections from different angles [3]. The advantage of DBT over “classical” 2D mammography is the reduction in the interference between regular breast tissue and possibly obscured tumors [4]. Several studies have shown an increased detection rate for breast cancer by adding DBT to conventional 2D mammography [4,5,6].

Some architectural distortions that are sometimes invisible in 2D mammography can be seen via DBT and can improve the discrimination between benign and malignant lesions [7,8].

Apart from architectural distortion, which is relatively often present in malignant lesions, other well-established diagnostic indicators for malignancy are conspicuous tumor tissue interface properties such as a diffuse border, spiculae, or larger ray-like changes to the surrounding tissue [9,10]. Well-circumscribed lesions are considered to be most likely benign, with only a small number of exceptions like papillary mucinous carcinomas, malignant phyllodes tumors, triple-negative tumors or cases with BRCA 1/2 mutations [11,12,13].

To improve the differentiation between benign and malignant lesions, some recent studies focused their attention on the peritumoral tissue/halo sign in DBT [14]. This sign describes a thin linear area of hyperlucency surrounding a circumscribed mass in mammography [15]. This artifact is a reason for the technical limitations of DBT. Due to the relatively small angular range of the tomography device, there is a limited sampling of the breast, and due to the physical properties of low-energy X-ray photons, they are preferentially attenuated when moving through very dense structures, which leads to beam hardening. This results in a low intensity shadow around high-density structures along the direction of the X-ray tube motion and/or sweep direction [16,17]. Although the exact cause of the halo is still not fully clarified, its appearance is considered a macroscopic marker for benign lesions in patients under 50 years of age [14], yet its diagnostic value for older patients is still argued [13].

To improve the management of screening detected possible benign breast lesions, the aim of this study was to investigate and compare the halo sign of breast lesions in DBT, with special attention to peritumoral linear alterations crossing the halo, termed the “broken halo sign”.

## 2. Materials and Methods

### 2.1. Approval by the Ethical Review Board (ERB)

Study approval was granted by the Ethical Review Board (ERB proposal 1109/2021).

### 2.2. Patient Inclusion and Case Classification

The patients were pre-selected in the application Centricity™ Radiology information systems “RIS-i 7” (General Electric Company, Boston, MA, USA), using a search mask to filter all patients who had received a breast biopsy at the hospital in the period from January 2019 to December 2021. The spectrum of selected patients was deliberately broad and included both screening and diagnostic mammography cases.

The inclusion criteria were (1) digital mammography including DBT performed at our department or an external practice with a modern mammography machine; (2) sufficient study quality following “Perfect, Good, Moderate, Inadequate” (PGMI) standards [18], including absence of motion artifacts, under- or overexposure or partial visualization of relevant pathologies; (3) available DBT volume in mediolateral oblique (MLO) projections; (4) available histology with a maximum interval of 3 months between DBT and biopsy.

The exclusion criteria were (1) patient age < 18 years, (2) ipsilateral breast cancer within the last 5 years, (3) artifacts affecting the DBT assessment, e.g., from breast marker clips, (4) mammographically inapparent tumors, (5) ipsilateral breast implant, (6) intracutaneous localization.

After screening of 288 cases, 191 female participants (64.1%) were included.

### 2.3. Technical Information

Following our center’s routine practice, all patients underwent 2D non-tomographic mammography in CC view and 3D DBT in MLO view. Tomosynthesis was performed on a Siemens Mammotom (Siemens Healthineers; Erlangen, Germany) or a Selenia Dimensions (Hologic; Marlborough, MA, USA). Image acquisition parameters such as compression, etc., followed international standards of the “American College of Radiology” (ACR). All tomosyntheses were performed in MLO orientation.

### 2.4. Image Interpretation and Data Collection

The DBT images were analyzed using our in-house picture archiving and communication system viewer IMPAX EE R20 XVIII (AGFA HealthCare; Mortsel, Belgium). The parameters assessed were the type of mammography device alongside the breast density according to the American College of Radiology (grades A–D) [19], the presence of calcifications including their shape (monomorphic, polymorphic, punctiform) and their arrangement (random, linear, grouped), any perilesional architectural alteration and the tumor surface configuration (smooth, lobulated, irregular).

**Figure 1:**
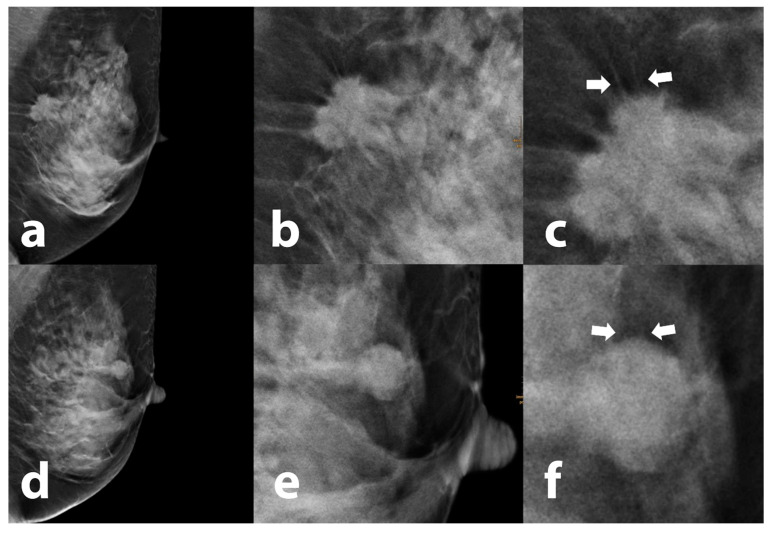
Overview and magnification of tumors exhibiting a peritumoral halo. Coarse tumor extensions (white arrows) cross a hypodense vertically oriented cap-like area surrounding an invasive breast carcinoma of “no special type” (NST) (**a**–**c**), while only subtle lines (white arrows) originate from the tumor surface from another histologically proven NST carcinoma (**d**–**f**). Even in areas with dense adjacent breast tissue, these fine surface irregularities are unmasked by the halo effect.

Halo presence and strength were graded as none, weak (mildly hypodense appearance) and strong (highly hypodense appearance). The halo circumference was defined as partial, circumferential or other (irregular halo not fitting in one of the former two categories). The halo shape was defined as diffuse, ring-shaped or oval. Linear hyperdensities entering or spanning the halo were classified as fine or broad (>0.5 mm) (Figure 1).

Finally, the focal lesion was measured, as well as the maximum size of the halo in the vertical image axis. Three exemplary individual measurements were taken in each case in representative areas of the halo.

Benign lesions such as cysts or intramammary lymph nodes discovered incidentally during screening were included in the study. Due to the lack of histological confirmation, these lesions were reported in stored sonography images and, if available, also in magnetic resonance imaging images. Only findings with clear agreement in the various imaging modalities were included in the evaluation.

### 2.5. Core-Needle Biopsy Procedure

Breast biopsies are routinely performed at our center. Following international guidelines [20], all patients with a lesion with a BI-RADS score of 4 or 5 are strongly advised to undergo an ultrasound-guided biopsy, if feasible. Additionally, some BI-RADS 3 lesions may also undergo a biopsy following clinical consideration. Patients with findings necessitating a stereotactic biopsy were not included in this analysis. The procedure was explained in detail to the patient and a signed informed consent was obtained. After skin disinfection, intracutaneous and ultrasound-guided perilesional application of a local anesthetic (Mepivacaine hydrochloride 1%), and a small skin incision, an automatic 12G or 14G core-needle biopsy system (HistoCore Automatic Biopsy System, BIP GmbH, Türkenfeld, Germany) was used to acquire five tissue specimens, which were then embedded in a 5% formalin solution for further staining and analysis.

### 2.6. Histologic Evaluation

After full imaging data collection, histopathological data were gathered from the hospital’s clinical information system KIS PowerChart (Cerner, North Kansas City, MO, USA).

Histological assessments were carried out by a specialized gynecological section of our local pathology institute following WHO guidelines [21]. If available, full resection specimens were used, if not, those from core-needle samples were used. If any form of neoadjuvant therapy had been administered since initial diagnosis, the histopathological core-needle biopsy reports were used instead.

### 2.7. Statistics

All data were stored in Microsoft Excel 16.16.21 (Microsoft; Redmond, WA, USA). The statistical software used was SPSS 27.0 for Windows (SPSS, Chicago, IL, USA) and GraphPad Prism 9.1.0 (GraphPad Software LLC.; La Jolla, CA, USA).

Descriptive statistics for all patients include demographic (age, breast density) and disease-related factors (tumor size, histological diagnosis, LN metastasis rate, hormone receptor positivity rate, Ki-67 index and HER2neu mutation rate). Results include mean ± standard deviation (SD) and ranges (in brackets) or relative frequency (absolute values in brackets).

Continuous data of the groups were compared via an ordinary one-way ANOVA with a Holm–Sidak correction or Kruskal–Wallis test with a Dunn’s post-test (in case of a non-Gaussian distribution, assessed by a D’Agostino and Pearson test) to correct for multiple testing. If a Gaussian distribution could be achieved through log-transformation, data were transformed for analysis. Categorical variables were compared via a pairwise Fisher’s exact test (in case of 2 × 2 tables) or a χ^2^ test. Statistical significance was considered for *p*-values < 0.05.

## 3. Results

### 3.1. Patient Characteristics

After screening of 288 cases, 191 female participants (64.1%) were included for further analysis. Ninety-seven patients had to be excluded due to breast implants (1.4%, *n* = 4), a mammographically inapparent tumor (26.0%, *n* = 75) or for other reasons (6.3%, *n* = 18). The average patient age at diagnosis was 58.9 ± 13.7 (range: 33.0 to 87.4 years). The breast density was on average rated as 2.4 ± 0.9 according to ACR (A = 1, B = 2, C = 3, D = 4) with ACR A in 17.9% (*n* = 34), ACR B in 33.7% (*n* = 64), ACR C in 34.2% (*n* = 65) and ACR D in 14.2% (*n* = 27) of cases.

### 3.2. Tumor Characteristics

Roughly one-third of cases yielded a benign result after biopsy (BI-RADS 2; 33.0%, *n* = 63), eight cases were probably of benign origin (BI-RADS 3; 4.2%) and 62.8% of cases were histologically confirmed as malignant (*n* = 120).

BI-RADS 2 lesions comprised mastopathy (39.1%, *n* = 18), fibroadenomas (39.1%, *n* = 18), focal mastitis or other focal inflammatory conditions (19.6%, *n* = 9), complicated cysts (17.9%, *n* = 10) and other diagnoses (12.5%, *n* = 7).

BI-RADS 3 lesions consisted of phyllodes tumors (75.0%, *n* = 6) and atypical ductal hyperplasia (ADH) (25.0%, *n* = 2).

Among BI-RADS 5 tumors, carcinomas of no special type (NST) (80.8%, *n* = 97) outweighed invasive lobular carcinoma (ILC) (6.7%, *n* = 8), ductal carcinoma in situ (DCIS) (4.2%, *n* = 5) and other types (8.3%, *n* = 10). Lymph node metastasis was present in 22.5% of cases with malignancies (*n* = 27). For more details, please refer to Table 1.

### 3.3. Tomosynthesis Evaluation

**Figure 2:**
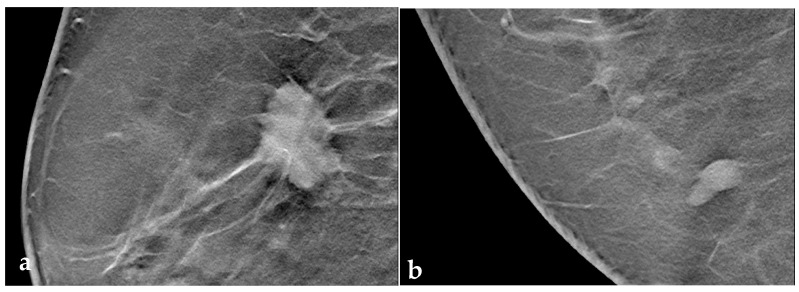
(**a**) Breast cancer of no special type (NST) with a strong peritumoral halo, demonstrating the surrounding architectural distortion, and also small spiculae crossing the halo zone (broken halo sign); (**b**) fibrous-cystic mastopathy with a weak, continuous cranio-caudally oriented halo without a horizontal component.

Overall, 82.2% (*n* = 157) of lesions demonstrated some kind of halo. BI-RADS 2/3 lesions had a weak halo in 33.8% (*n* = 24) and strong halo in 40.8% of cases (*n* = 29). In the BI-RADS 5 group, 44.2% (*n* = 53) had a weak halo and 42.5% (*n* = 51) had a strong halo. A broken halo sign was visible in 38.4% of benign lesions and in 89.4% of malignant lesions (see Table 2 and Figure 2).

In all study participants, regardless of breast density, the presence of a broken halo was a significant indicator of malignancy with an OR of 6.33 (95% CI: 2.51 to 15.95) and an irregular tumor surface (OR 5.49, 95% CI: 1.64 to 18.4) (see Table 3).

In contrast, in patients with higher breast densities (ACR C and D), the broken halo was the only significant criterion for malignant differentiation (see Table 4).

**Figure 3:**
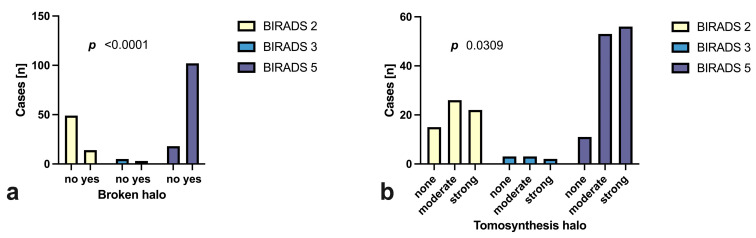
Presence and strength of a tomographic halo (**a**) and presence of the “broken halo” sign (**b**) grouped by histological BI-RADS result.

There was a significant difference concerning the tomographic halo occurrence rate (*p* < 0.0001) and strength (*p* = 0.031) by BI-RADS group (Figure 3a,b). Furthermore, a “broken halo sign” was found in the majority of BI-RADS 5 tumors (77.5%, *n* = 93), in 50.0% of BI-RADS 3 tumors (*p* < 0.0001) and in 27.0% (*n* = 17) of benign BI-RADS 2 tumors (Figure 3b).

**Figure 4:**
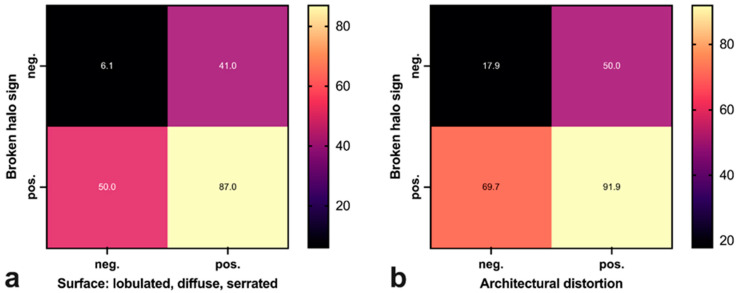
Malignancy rates for the combination of broken halo and (**a**) a “non-smooth surface” (lobulated, diffuse, serrated) and (**b**) an “architectural disturbance”, shown as a heat map.

Combined with irregular tumor surface (Figure 4a) or architectural distortion (Figure 4b), the presence of a broken halo sign was more specific of malignancy, yet it did not increase the overall correct classification rate (CCR). For further diagnostic properties of the broken halo and other tomographically determinable variables such as size, surface morphology and architectural distortion, please refer to Table 5.

## 4. Discussion

Traditionally, the halo sign is defined as a radiolucent ring a few millimeters wide that at least partially surrounds a focal lesion. Traditionally, it has been described to appear around lesions with a smooth contour such as cysts or fibroadenomas, and has been considered an indicator of benignity [14]. However, over the last decade, the halo sign has increasingly been acknowledged as also occurring around malignant tumors [13,15]. To complicate things further, some breast malignancies such as triple-negative cancers or those with BRCA mutations may exhibit both a smooth border and a halo and may therefore be falsely diagnosed as benign [12,13,22].

Several hypotheses concerning the presence of a perilesional halo have been discussed over the years, including compressed fat tissue [23], a perceptual illusion (Mach band) [24] or technical limitations of DBT [16,17]. Although the cause of the halo sign still remains unclear, the presence of a halo sign can be a helpful indicator of macroscopically smooth lesion margins and may contribute to increased contrast between a circumscribed mass and surrounding breast tissue in DBT. While the consensus is that circumscribed lesions with a halo can be considered benign on 2D mammograms, this is not the case for circumscribed lesions in DBT, as they can neither be benign or malignant [13]. Those findings are in accordance with the findings in this report, as the halo frequency and strength did not differ significantly between BI-RADS groups (*p* = 0.216).

According to the literature [9,10], lesions with indistinct margins or irregular shape are suspicious and need further evaluation. However, especially in elderly women, even breast lesions with smooth circumscribed margins are malignant in 8–9% of cases (8.8% according to Xiu et al. [25], 9.0% according to Liberman et al. [10]). Accordingly, a circumscribed tomosynthesis border is no guarantee for a benign lesion [26]. Furthermore, such irregular margins may be obscured by adjacent dense breast tissue, be it either focal areas of denser parenchyma or a general high parenchymal density (ACR density C and D).

A study by Sánchez-Camacho González-Carrato et al. [14] showed that a tomosynthesis halo sign was a good diagnostic predictive marker of benign lesions in women <50 years old, but not for women >50 years old. As many of the patients with breast cancer are 50 years or older, the halo sign as a benign marker only accounts for a small percent of patients. In our study, the mean patient age was ~58 years and only 13.3% of the BI-RADS V lesions showed no halo, whereas the rest of them showed a more or less pronounced one.

Peritumoral tissue undergoes several changes [27,28], reflecting a more or less spatially organized “ecosystem”, aptly discussed by Sofopoulos et al. [29]. Desmoplastic reactions can occur as a response of the body against malignant tumors [30]; among these, periductal fibrotic reactions [31] may lead to a contraction of the peritumoral tissue. Besides these fibrotic changes, malignant tumors affect the microvascular environment via the VEGF pathway [32]. These changes may lead to the appearance of a serrated border or prominent tumor extensions upon mammography and DBT, and may also cause the appearance of variably oriented halos. Thus, the “many smaller” haloes of the offshoots, together with the actual halo of the lesion itself, could form a composite artifact that appears to completely surround the focal lesion to generate a more pronounced overall halo.

In addition to the overall evaluation of a possible DBT halo, peritumoral tissue changes may also lead to minuscule septations or lines within the halo, which we termed a “broken halo” sign. This sign showed the highest PPV of all criteria examined in our study in the evaluation of the peritumoral tissue. Since there are unfortunately no recent works that deal with extratumoral changes in tomography in a similar way to ours, we compared our work with the work by Xu et al., who described the extratumoral changes in digital breast mammography in a similar way [25]. They described a “vertical trabecula sign” in the digital mammography in the MLO and CC view, which in some way is comparable to our “broken halo” sign, with a PPV of 83.6%. The “broken halo sign” had a PPV of 85.7%. Besides the “vertical trabecula sign”, they also mentioned a high PPV for their so called “extratumoral contraction sign”, at 96.6% [25]. The “extratumoral contraction sign” is most likely comparable to the architectural distortion in our study, but in contrast to the high PPV of 96.6% determined by Xu et al. [25], the PPV of our architectural distortion alone was somewhat lower at 85.3%. Still, the combination of the “broken halo sign” and the architectural distortion had a similar PPV to the “extratumoral contraction sign” at 91.9%.

Interestingly, an analysis of cases with a higher breast density (ACR C, D) showed that most of the common predictors for malignancy analyzed above lost diagnostic power, with the only significant parameter to identify malignancy based on the peritumoral changes in this study being the broken halo sign. Due to the tight contact of tumoral and peritumoral glandular tissues, lesions in dense breasts without strong accompanying architectural distortions may remain difficult to detect even in DBT. Even though DBT generally has an increased detection rate of malignant lesions, the visualization of small architectural disturbances in dense breast tissue may not be possible due to the drawbacks of low-dose X-ray projections (electronic noise, low contrast, etc.) [3,13].

The halo around high-contrast objects as well as around focal lesions is not constant and can be completely absent. This may be due to the location of the lesion, as in DBT there is a known “blurring ripple” artifact, which is described as a blurred transition of high contrast objects to the surrounding breast parenchyma dependent on the location. When the high-density objects are visualized in sections immediately out of plane, they occur with a blurred transition, whereas if they are in the center, the “blurring ripple” artifact is not visible [33]. Due to this blurred transition, the halo may be distorted and the small changes influencing the “broken halo sign” may vanish in these artifacts.

**Figure 5:**
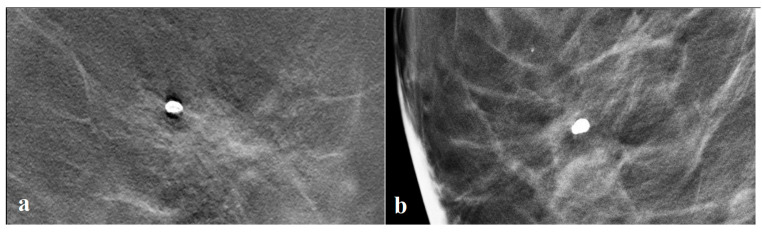
(**a**) Strong halo around a dense focal calcification by vendor A (Mammomat, Siemens, Erlangen, Germany); (**b**) no halo around a similar calcification on a mammography machine by vendor B (Selenia Dimensions, Hologic, Marlborough, MA, USA).

Besides the “blurring ripple” artifact, the mammography device also plays an important role. Depending on the vendor and machine hardware and software, similar focal lesions may not produce the same strength halo artifact (Figure 5a,b).

One explanation could be that the reconstruction algorithms of some manufacturers reach their limits at the diffuse edges of certain lesions; it may be technically easier to suppress this artifact at sharply defined edges. Also, the location of the lesion in the field of view may play an important rule.

In addition to the recording parameters that vary between manufacturers (e.g., the tomosynthesis angle), the software used may play an even more decisive role. Wu et al. [34] examined this by using different reconstruction algorithms and could demonstrate a strong influence of these algorithms on halo artifacts.

### Limitations

This study has several limitations. Due to the fact that primarily suspicious lesions are biopsied for further clarification, benign findings were relatively underrepresented due to a lack of corresponding histological results. Assuming that the tomosynthesis halo is an artifact that occurs during the acquisition or reconstruction process, even more comprehensive consideration of the mammography device used, including specific settings and patient-related parameters such as compression, would be necessary, as only two different tomography systems are in routine diagnostic use. However, regardless of the expression of the halo sign, the “broken halo sign”, if a halo was visible, was comparable between both devices. Within the scope of this study, most imaging data originated from two manufacturers. The influence of recording parameters or the software used on the halo presence and configuration cannot be ruled out. Further developments of the DBT technique, including successful suppression of such halo artifacts, could lead to a loss of the broken halo sign. To a certain extent, the broken halo that was researched in this study as a reliable indication of a malignant finding would then be lost.

For possible future investigations in this area, it would be advisable to consider the “halo” in DBT depending on the peritumoral changes and even the smallest linear interruptions in the “halo” itself. The breast density was indeed recorded as part of this study, but this reflects the entire breast, and any possible regional differences were not taken into account. Neighboring, very radiopaque structures could possibly influence the tomosynthesis halo.

## 5. Conclusions

The broken halo sign—the presence of small lines of variable diameter spanning peritumoral areas of hypodensity—appears to be a valuable differentiator of malignant breast lesions. This is especially true in denser breasts (ACR C/D), where architectural distortion may be masked. Further studies are needed to evaluate this imaging marker on a wider set of mammography machines and reconstruction algorithms.

## Figures and Tables

**Table 1:** Overview of histological lesion results.

	Overall (%, *n*)	Histological Diagnosis (%, *n*)	Grade (%, *n*)
BI-RADS 2	33.0 (63)	Mastopathy: 39.1 (18)	
		Fibroadenoma: 39.1 (18)	
		Focal mastitis/inflammatory: 19.6% (9)	
		Complicated cyst: 17.9% (10)	
		Other: 12.5% (7)	
BI-RADS 3	4.2 (8)	Phyllodes tumor: 75.0% (6)	
		Atypical ductal hyperplasia (ADH): 25.0% (2)	
BI-RADS 5	62.8 (120)	No special type (NST): 80.8% (97)	Grade 1: 16.5 (16)
			Grade 2: 59.8 (58)
			Grade 3: 23.7 (23)
		Invasive lobular carcinoma (ILC): 6.7% (8)	Grade 1: 0.0 (0)
			Grade 2: 87.5 (7)
			Grade 3: 12.5 (1)
		Ductal carcinoma in situ (DCIS): 4.2% (5)	low-grade: 20.0 (1)
			high-grade: 80.0 (4)
		Other: 8.3% (10)	Grade 1: 50.0 (5)
			Grade 2: 40.0 (4)
			Grade 3: 10.0 (1)

**Table 2:** Halo visibility and broken halo sign grouped by BI-RADS categories.

	Halo Visibility: % * (*n*)			Broken Halo Sign: % * (*n*)
BI-RADS 2/3	no halo:	25.4	(18)	39.4 (21)
	weak:	33.8	(24)	
	strong:	40.8	(29)	
	∑	100	(71)	
BI-RADS 5	no halo:	13.3	(16)	89.4 (93)
	weak:	44.2	(53)	
	strong:	42.5	(51)	
	∑	100	(120)	

**Table 3:** Multivariate analysis of predictors for malignancy in all patients (ACR A–D).

	Regression Coefficient B	Standardized Error (SE)				
			*p*-Value	OR	Lower	Upper
Architectural distortion (yes)	1.251	0.497	0.012	3.493	1.319	9.247
Maximum size	−0.029	0.018	0.107	0.971	0.937	1.006
Maximum halo depth	0.054	0.061	0.375	1.056	0.937	1.190
Broken halo (yes)	1.846	0.471	0.000	6.331	2.513	15.952
Conspicuous margin (yes)	1.702	0.618	0.006	5.485	1.635	18.400
Shape (irregular)	0.997	0.571	0.081	2.709	0.885	8.296

**Table 4:** Multivariate analysis of predictors for malignancy in all patients (ACR 1–4).

	Regression Coefficient B	Standardized Error (SE)				
			*p*-Value	OR	Lower	Upper
Architectural distortion (yes)	1.059	0.627	0.092	2.882	0.843	9.860
Maximum size	−0.041	0.024	0.092	0.960	0.916	1.007
Maximum halo depth	0.010	0.102	0.920	1.010	0.828	1.233
Broken halo (yes)	1.653	0.769	0.031	5.224	1.158	23.564
Margin (diffuse)	2.025	1.178	0.086	7.578	0.753	76.239
Shape (irregular)	1.190	0.856	0.164	3.286	0.614	17.590

**Table 5:** Diagnostic properties of tomographic lesion variables.

Predictor	Sensitivity (%)	Specificity (%)	PPV (%)	NPV (%)	Likelihood Ratio	CCR (%)	*p*-Value
Max. tumor diameter (cut-off 10.8 mm)	88.3 (81.4 to 92.9)	26.8 (17.9 to 38.1)	67.1 (59.4 to 73.9)	57.6 (40.7 to 72.8)	1.21	65.5	0.0100
Architectural distortion	72.5 (63.9 to 79.7)	78.9 (68.0 to 86.8)	85.3 (77.2 to 90.9)	62.9 (52.6 to 72.6)	3.43	74.9	<0.0001
Tumor surface (lobulated, diffuse, serrated)	96.7 (91.7 to 98.7)	46.5 (35.4 to 58.0)	75.3 (68.9 to 81.5)	89.2 (75.3 to 95.7)	1.81	78.0	<0.0001
Broken halo sign	85.0 (77.5 to 90.3)	76.1 (65.0 to 84.5)	85.7 (78.3 to 90.9)	75.00 (63.9 to 83.6)	3.55	81.7	<0.0001
Broken halo + architectural distortion	65.8 (57.0 to 73.7)	90.1 (81.0 to 95.1)	91.9 (84.1 to 96.0)	61.0 (51.4 to 69.7)	6.677	74.9	<0.0001
Broken halo + conspicuous tumor surface (lobulated, diffuse, serrated)	83.3 (75.7 to 88.9)	78.9 (68.0 to 86.8)	87.0 (79.6 to 91.9)	73.7 (62.8 to 82.3)	3.94	81.7	<0.0001

## Data Availability

Data will be available on request to authors.

## Abbreviations

The following abbreviations were used in this manuscript:

IC	interval cancer
CI	confidence interval
ACR	American College of Radiology
IBC	interval breast cancer
NST	no special type
ILC	invasive lobular carcinoma
DCIS	ductal carcinoma in situ
LN	lymph node
RIS	radiology information systems
PGMI	Perfect, Good, Moderate, Inadequate
MLO	mediolateral oblique
